# Early Neonatal Death Possibly From a Rare Congenital Coronary Artery Anomaly

**DOI:** 10.7759/cureus.29671

**Published:** 2022-09-27

**Authors:** Pradeep Kumar Velumula, Erica N Leclair, Obeid M Shafi, Samir Chandra

**Affiliations:** 1 Pediatrics and Neonatology, Mercy One Waterloo Medical Center, Waterloo, USA; 2 Pediatrics, Arkansas Children's Hospital, Little Rock, USA; 3 Clinical Informatics, University of Arkansas for Medical Sciences, Little Rock, USA; 4 Cardiology, Mercy One Waterloo Medical Center, Waterloo, USA

**Keywords:** term neonates, cardiac arrest in neonates, early neonatal death, left coronary artery ostium stenosis, newborn and child health

## Abstract

We report a term female newborn who presented with bradycardia and weak respiratory efforts immediately after birth. Mother had an uneventful pregnancy and the infant was delivered by cesarean section secondary to arrest of labor. The infant did not respond to the neonatal resuscitation and was declared dead 32 minutes after birth. Autopsy findings include left coronary artery (LCA) ostium stenosis and moderate-to-severe chorioamnionitis on placental examination. An autopsy did not find any anatomic or histologic abnormalities in other organ systems that could be attributed to the cause of early neonatal death. To the best of our knowledge, ours is the third case reported in the literature on LCA ostium stenosis presenting immediately after delivery. Unfortunately, all the infants had a fatal outcome. Our case report emphasizes the importance of a meticulous autopsy examination, considering coronary artery anomalies, in case of early neonatal deaths.

## Introduction

In the uterus, a fetus is in a relatively hypoxic environment and has a right-ventricle-dominant circulation. Due to the presence of a low-resistance placenta, the systemic vascular resistance in the fetus is low compared to the pulmonary vascular resistance. The right ventricle predominantly supplies the lower part of the body through the patent ductus arteriosus and the left ventricle supplies the upper part of the body. At birth, as the umbilical cord is clamped and the low-resistance placenta is cut off, the workload on the left ventricle is increased due to an increase in the systemic vascular resistance along with an increased amount of blood return from the lungs [1]. In newborns with left coronary artery (LCA) ostium stenosis, the physiological changes at birth along with the increased workload on the left ventricle could possibly lead to left heart failure and death. LCA ostium stenosis is an extremely rare condition with very few cases being reported in the literature [2].

## Case presentation

The infant's mother is 20 years old, gravida1 para0, and was admitted for induction of labor for late-term pregnancy at 41 0/7 weeks gestational age. She had regular prenatal care and was taking prenatal vitamins during her pregnancy. She had no significant past medical and surgical history. Prenatal laboratory work was negative for HIV, hepatitis B surface antigen, syphilis, gonorrhea, chlamydia, and Group B Streptococcus. She was followed up by the maternal-fetal medicine department for a marginal cord insertion and had normal fetal growth patterns and fetal surveillance during the pregnancy. During the labor, there were no concerns with fetal heart rate tracing. She had a temperature of 38.5 degrees Celsius 2 hours before the delivery and was started on antibiotics for suspicion of chorioamnionitis. The infant was delivered by cesarean section, under epidural anesthesia, secondary to arrest of descent of fetal head. Immediately after delivery, the infant had a weak cry with irregular respiration and some flexion of extremities. While delayed cord clamping was performed, suction of the mouth and the oral cavity was done, which was followed by stimulation. APGAR scores at 1 and 5 minutes after birth were 3 and 1, respectively.

The infant was handed over to the neonatal intensive care unit (NICU) team at ~1 minute after birth. Due to weak respiratory efforts and heart rate <100 beats/minute, following the initial steps of resuscitation, positive pressure ventilation was initiated with a bag and mask and later through an endotracheal tube. Neonatal resuscitation was performed as per Neonatal Resuscitation Program guidelines, edition 8. Despite providing the resuscitative efforts with positive pressure ventilation, infants' heart rate dropped to <60 beats/minute when chest compressions were initiated, and an emergency umbilical venous line was placed. In total, she received one dose of epinephrine through the endotracheal tube and four doses of intravenous epinephrine through an umbilical venous line. She also received one normal saline bolus, 10 mL/kg, during the resuscitation. At 32 minutes after birth, she was declared dead. Her heart rate was less than 100 beats/minute throughout the resuscitation.

Umbilical cord arterial blood gas analysis showed a pH of 7.21 and a base excess of negative 7.1. Her birth weight was 3.39 kg (49%ile). No other laboratory work was performed on the infant. Autopsy of the infant noted LCA ostium stenosis and moderate-to-severe chorioamnionitis on placental examination. The autopsy did not show any anatomic or histologic abnormalities in other organ systems that could be attributed to the cause of death.

## Discussion

The infant illustrated in this report had an early neonatal death possibly from cardiac failure secondary to LCA ostial stenosis. In the uterus, the fetus is in a relatively hypoxic environment, has a low systemic vascular resistance, and is with predominant right ventricle circulation. The oxygenated blood from the placenta reaches the coronary arteries, bypassing the pulmonary circulation, through the foramen ovale. Also, the fetal and newborn cardiac muscle works at the peak of frank starlings’ law with minimal reserve [1]. In the setting of LCA ostium stenosis, we presume, the reasons for failed adaptation in the neonate reported could be 1) increased oxygen demand of the left ventricle after birth, secondary to increased systemic vascular resistance, 2) insufficiently developed collaterals, 3) stress from the labor, 4) and maternal chorioamnionitis. The above-mentioned causes could have impaired the left ventricular function in the infant leading to failed adaptation to the extrauterine life and immediate neonatal death. The autopsy report showing no anatomic and histologic abnormalities in any other organ systems further strengthens our hypothesis. The histopathology of the heart did not show any signs of chronic ischemic changes, implying an acute event after delivery.

LCA ostial stenosis is an extremely rare congenital anomaly of the coronary arteries [2]. There is very limited evidence in the published literature, from case reports and case series, about the condition, its outcomes, and the long-term prognosis [3-5]. The anomaly can present as an isolated lesion or can present along with other cardiac anomalies [5-7]. Cases have been reported with varied clinical presentations from infancy to adulthood [8]. Infants and children frequently presented with sudden cardiac failure or with respiratory distress symptoms secondary to heart failure, while adolescents and adults presented with more variety of symptoms like sudden cardiac arrest, syncope, cardiac rhythm abnormalities, myocardial infarction, and angina [5,8-11]. Daniela et al. have published a case series of five neonates who presented with this anomaly in the neonatal period. Of these five neonates, two neonates presented immediately after delivery and had a fatal outcome. To the best of our knowledge, this is the only case series in the published literature with the neonatal presentation of LCA ostium stenosis [10]. Some patients from the previous case reports were misdiagnosed as having an anomalous origin of the left coronary artery from the pulmonary artery (ALCAPA) and found to have LCA ostium stenosis or atresia on the operative table [11,12]. ALCAPA, another coronary artery anomaly, has similar clinical manifestations and is more common than LCA ostium stenosis or atresia.

Coronary angiography is the diagnostic method of choice to diagnose LCA ostium stenosis/atresia. Cardiac CT imaging is being used in adults and children to recognize coronary artery anomalies but its use in infants and young children is limited due to a lack of evidence [13,14]. Neonates diagnosed with LCA ostium stenosis from the published case series have all died similar to the neonate described in our report [10]. In older children and adults, revascularization has been achieved using modalities like coronary artery bypass graft, surgical angioplasty, and osteoplasty [5,11,15,16]. Long-term prognosis cannot be ascertained as there are not enough studies looking into it due to the rarity of its condition.

## Conclusions

LCA ostial stenosis/atresia is a rare coronary artery anomaly that can cause early neonatal death. Though rare, it should be considered in the differential diagnosis in infants and children presenting with sudden cardiac arrest and respiratory distress secondary to heart failure. Our case report also emphasizes the importance of a meticulous examination during an autopsy, considering coronary artery anomalies, in case of early neonatal deaths. 
